# Supplementary material for: Towards unified AI-driven fracture mechanics: the extended deep energy method (XDEM)
Source: Nat Commun. 2026 Aug 15;17:8492. doi: 10.1038/s41467-026-76748-1 (PMC13484471; doi:10.1038/s41467-026-76748-1)
Supplement: Supplementary file 3 [file 41467_2026_76748_MOESM3_ESM.zip › pic/Results/Stress_intensity_factor/Mode2/k2_comparison_different_tau.pdf]

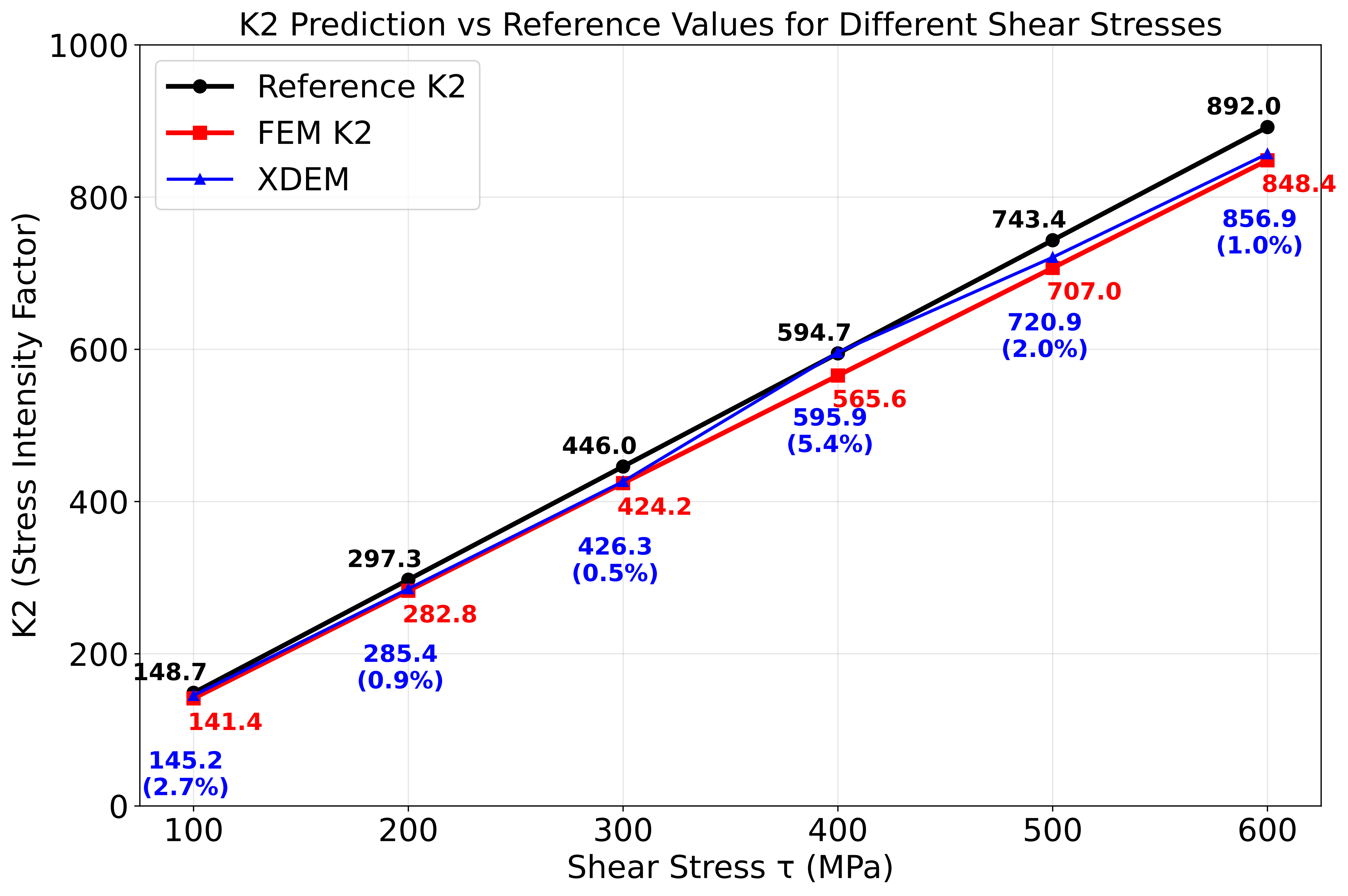
K2 Prediction vs Reference Values for Different Shear Stresses
K2 (Stress Intensity Factor)
Shear Stress τ (MPa)
Reference K2
FEM K2
XDEM
1000
800
600
400
200
0
100
200
300
400
500
600
148.7
141.4
145.2
(2.7%)
297.3
282.8
285.4
(0.9%)
446.0
424.2
426.3
(0.5%)
594.7
565.6
595.9
(5.4%)
743.4
707.0
720.9
(2.0%)
892.0
848.4
856.9
(1.0%)
